# Clusters, lines and webs—so does my patient have psychosis? reflections on the use of psychiatric conceptual frameworks from a clinical vantage point

**DOI:** 10.1186/s13010-022-00118-0

**Published:** 2022-02-14

**Authors:** Tibor Zoltan Kovacs, Reece William Hill, Stuart Watson, Douglas Turkington

**Affiliations:** 1 Early Intervention in Psychosis Service, Newcastle upon Tyne, Cumbria UK; 2 Northumberland Tyne and Wear NHS Foundation Trust, 1 Benton View, Forest Hall, Newcastle upon Tyne, NE12 7JJ UK; 3 Translational and Clinical Research Institute, Newcastle University, Newcastle upon Tyne, NE1 7RU UK; 4 School of Medical Education, Newcastle University, Framlington Place, Newcastle upon Tyne, NE2 4HH UK; 5 Translational and Clinical Research Institute, Newcastle University, Newcastle upon Tyne, NE1 7RU UK; 6 Cumbria, Northumberland, Tyne and Wear NHS Trust, Monkwearmouth Hospital, Newcastle Road, Sunderland, SR5 1NB UK

**Keywords:** Psychosis, Schizophrenia, Diagnosis, Categorical, Dimensional, Network, Essentialism, Schema, Framework

## Abstract

Mental health professionals working in hospitals or community clinics inevitably face the realisation that we possess imperfect conceptual means to understand mental disorders. In this paper the authors bring together ideas from the fields of Philosophy, Psychiatry, Cognitive Psychology and Linguistics to reflect on the ways we represent phenomena of high practical importance that we often take for granted, but are nevertheless difficult to define in ontological terms. The paper follows through the development of the concept of psychosis over the last two centuries in the interplay of three different conceptual orientations: the categorical, dimensional and network approaches. Each of these represent the available knowledge and dominant thinking styles of the era in which they emerged and take markedly different stances regarding the nature of mental phenomena. Without particular commitment to any ontological positions or models described, the authors invite the reader into a thinking process about the strengths and weaknesses of these models, and how they can be reconciled in multidisciplinary settings to benefit the process of patient care.

## Introduction

Psychiatric diagnosis deals with abstract concepts developed to capture the nature of mental disorders in all their multi-layered and multi-faceted complexity. Consequently, everyday work in mental health presents clinicians with questions that cannot be answered without a certain degree of philosophical enquiry. Although clinicians often tend to brush away a theoretical stance reaching for ‘practical’ solutions, all too often one gets stuck in the middle of a decision-making process in the absence of satisfying answers to fundamental questions. One such question is *“Does my patient suffer from psychosis?”,* one that begs for answer at certain crucial bifurcations of care pathways, such as when deciding whether to provide care in a service specialized in psychosis (e.g. Early Intervention in Psychosis Teams) and whether to start antipsychotic medication. These questions can raise significant tensions in multidisciplinary settings where professionals with different training backgrounds often differ in their conceptual orientation and (implicit or explicit) views regarding the nature of mental disorders. Recognising the origins, merits and limitations of multiple views in this context therefore becomes a necessary aspect of care-in-action.

In this paper we examine the main diagnostic approaches in the light of philosophical, historic, psychological and linguistic considerations, aiming to draw attention to potential biases and assumptions inherent to their specific ways of thinking. Due to its recent release and important changes in its conceptual orientation, we will focus mainly on ICD-11 [1]. The example of psychosis is used as a specific case against the backdrop of more general considerations regarding disorders with complex aetiologies.

As scientific methodologies progress, the complexity of the field seems to increase along with the number of contributing factors we know about. To complicate matters further, the development of a diagnostic toolkit always unfolds in a specific historic context with characteristic social, political and financial influences. Therefore, it continues to be vulnerable to ‘the four idols’ of human thought that Francis Bacon warned us about 400 years ago in his *Novum Organum* ([2], p. 22). He stated that “*The human understanding, from its peculiar nature, easily supposes a greater degree of order and equality in things than it really finds; and although many things in nature be sui generis and most irregular, will yet invent parallels and conjugates and relatives, where no such thing is*.”. The first type, the ‘idols of the tribe’ refer to biases universally shared by the entire human race as, Bacon asserts, “*all the perceptions both of the senses and the mind bear reference to man and not to the universe*”. The ‘idols of the den’ refer to the influences of the particular kind of education, readings and the authority of previous scholars we encounter throughout our development. Using the term ‘idols of the market’ Bacon brings into our awareness the influence of words, concepts we use to interact, or ‘trade’ ideas with. If not ‘properly’ grounded in sound method and broad, gradual experimentation, words can create “*a wonderful obstruction of the mind*”[[2], p. 21] and “*manifestly force the understanding*”, thus leading to fallacies. Lastly, the ‘idols of the theatre’ warn against the blind influence of existing systems of thought, practices, and traditions “*as so many plays brought out and performed, creating fictious and theatrical worlds*” [[2], p. 22].

### The metaphysical dilemma of mental disorders

Views regarding the ontological reality of mental disorder have gone through remarkable changes over the last two centuries, mirroring developments in the Philosophy of Science. Some theories assume that the defining features (the ‘essence’) of mental disorders are rooted in biology, seeking for anatomical localisations, genes, and neural pathways (biological essentialism), while others define mental disorders as disturbances of psychological constructs such as self-experience, attachment, theory of mind (phenomenological and psychological essentialism). Still others try to completely break away from essentialism and seem to glimpse mental disorder residing solely in the web of interactions (network approach).

Reflecting on the ontological ‘kinds’ of heterogeneous observations Haslam identifies several ‘kinds of kinds’ [3], including ‘natural kinds’ (where biological aetiology is known), ‘discrete kinds’ (clearly defined syndromes), ‘practical kinds’ (categories with arbitrary boundaries), ‘fuzzy kinds’ (with no clear boundaries) and ‘non-kinds’ (e.g., dysfunction defined along several dimensions). He points to separate disorders that exemplify each of these kinds, arguing for a pluralistic view in diagnosis. This diversity of kinds is clearly noticeable in the diagnostic entities of ICD-11, as these have different time courses, different number of symptom domains and different numbers of co-occurring syndromes. Although this diversity could be criticised as a weakness, it seems likely that it merely reflects the complex and heterogenous nature of the phenomena. Recognising the difficulty of defining essential entities, the fundamental wisdom of diagnostic classifications remains that—as suggested by Schwartz and Wiggins [4] – “*Psychiatry is a practical science”*; it is driven by values such as promoting health and alleviating suffering. Consistent with this pragmatic approach, our classification systems adopt the position of scientific realism, endorsing belief in the existence of both observable and unobservable entities, provided they are reasonably defined and supported by the scientific enquiry.

### The elusive concept of psychosis

The concept of *psychosis* initially emerged in the early nineteenth century, at the time when mental illness started to be differentiated from other social deviances [5] and from other medical conditions. Initial definitions followed a virchowian localisationist tradition, assuming some forms of neurological lesion in the background. Cantstatt, perhaps the first to introduce the concept in 1841, used it synonymously with the term ‘psychic neurosis’ to delineate all those conditions affecting the nervous system that have primarily ‘psychic’ (psychological) manifestations [6]. However, the lack of histological lesions led some authors to question the ‘reality’ of some forms of psychoses. For example, Alois Alzheimer made the distinction between ‘real psychoses’ (such as dementia paralytica and dementia praecox) and ‘functional psychoses’ (including manic-depressive insanity), emphasising (in contrast with Franz Nissl) that not all psychoses could be linked to cortical pathological findings [7]. In the absence of visualised lesions Kahlbaum and Kraepelin made the leap from the materialist to the conceptual realm, postulating the existence of hypothetical entities (diagnostic constructs) that capture ‘the essence’ of different conditions [8] through systematic description of patient experience, behaviour and of the longitudinal course of disorders.

### Categorical approaches

Creating mental categories based on similarities is one of the most efficient and ubiquitous tools of human cognition [9]. Categories enable us to abstract, register and transfer knowledge, exponentially increase cognitive economy and our efficiency to deal with large numbers of individual situations. Learning based on categories is present very early in human development and is reinforced throughout our daily, common-sense interactions and by the academic learning process [10].

In *Categories* [11]*,* one of the core writings of Western Philosophy, Aristotle provides an examination of how things occur as separate entities in thought and how we refer to them in language. His system is firmly grounded in the natural kinds of things (substances) but includes categories such as ‘quality’, ‘quantity’ and ‘relations’. In addition to this theoretical construct, he created the first systematic study of the animal world based on observation of anatomical forms and functionality.

One of the great triumphs of categorical thinking is the development of medical taxonomies in response to an ardent need for public health reform, as pointed out by Florence Nightingale in the Fourth International Statistical Conference, London (1860). Her proposal, in line with similar efforts in Continental Europe and the USA established the foundations of the International Statistical Classification of Diseases and Related Health Problems [12].

The early psychiatric classifications developed by Kahlbaum and Kraepelin were firmly rooted in the biological essentialist tradition [13, 14], partly inspired by Carl Linneus, who published his *Systema Naturae* in 1735. It is not a coincidence that Emil Kraeplin’s older brother, Karl Kraepelin was a biologist who created an exhaustive taxonomy on the order of Scorpiones, and it was suggested that Karl might have encouraged his younger brother to develop a classification system for mental illnesses [15]. After his initial differentiation of psychosis into ‘dementia praecox’, ‘manic-depressive insanity’ and ‘other psychoses’ the concept of psychosis became detached from affective disorders and other presentations, being applied only to conditions characterised by some degree of reality distortion. However, these conditions have always shown a great deal of heterogeneity, which was captured in further subcategories such as paranoid, hebephrenic, catatonic and simplex sub-forms. After the term ‘schizophrenia’ was coined by Eugen Bleuler in 1911 [16], it has become an umbrella term synonymous with almost all non-organic psychoses, attracting the vast majority of efforts in terms of research and conceptual definition.

More recently statistical methodologies have been used to identify symptom groups that ‘hang together’—namely cluster analysis and discriminant factor analysis [17–19] - with some authors linking symptom clusters with putative biological causation i.e., cortical thickness [20, 21].

In contrast with this predominantly biological orientation stands the phenomenological approach developed by Karl Jaspers. He proclaims that “*man is not confined to what is biologically known of him*” ([22], p. 559) and advocates for a holistic understanding of a person in their cultural and experiential reality. Jaspers introduces ‘ideal types’ as a phenomenological essentialist approach “*to give structure to the transient manifold*” ([22], p. 560). Following Jaspers definition, Schwartz and Wiggins [4] argue that the identification of ideal types in psychopathology is not only *“a matter of simple averaging”* but captures essential properties of anomalous experiences via phenomenological analysis.

A computationally oriented approach that does not require the assumption of essential ‘ideal types’ is represented by Eleanor Rosch and Amos Tversky [23, 24], among others, in their general theory of categorical thinking. According to them, we think of category membership in probabilistic ways as correlational prototypes, trying to “*reduce the infinite differences among stimuli to behaviourally and cognitively usable proportions*”. Based on these processes, we judge objects (i.e., clinical presentations) as being more or less ‘representative’ or ‘prototypical’ examples of a given category (i.e., disorder), based on the number and validity of features they carry (diagnostic criteria) an application of this approach to Psychiatry is described by Westen [25].

Critical discussions of the dominant categorical diagnostic systems (ICD-10 and DSM-5 [26]) pointed out that although these systems increased diagnostic reliability, the validity of the categories is not satisfactorily established [27, 28]. Categories tend to have ‘fuzzy’ boundaries and it is often difficult to tell whether a presentation belongs to one or another category; classic illustrations of this point include the complex interface between psychotic and mood disorders, as well as between mood and anxiety disorders, leading to diagnostic constructs such as ‘Schizoaffective disorder’ and ‘Mixed depressive and anxiety disorder’. Kendell and Jablensky recommend that in order to identify a valid syndrome, this needs to be separated from other syndromes and from normality by a ‘zone of rarity’ around the edges and needs to be defined by some natural characteristics beyond ‘superficial’ descriptive features (i.e., symptoms, course and outcome) [28]. However, such distinctive, defining characteristics are rarely established with any clarity. Due to this rationale the ICD-11 abandoned traditional sub-categories of schizophrenia, finding insufficient evidence for their predictive or treatment validity, and adopted instead a set of dimensional symptom specifiers (positive, negative, depressive, manic, psychomotor, and cognitive symptoms) rated on a four-point severity scale applied across all diagnostic categories in the group ‘Schizophrenia or other primary psychotic disorders’ [29]. This approach represents a major rupture both from the ideal type and prototype approaches, as different patients diagnosed with schizophrenia may well display entirely different symptom profiles, leaving the overarching conceptual construct difficult to grasp.

### Dimensional approaches

Dimensional frameworks identify psychotic disorders on a ‘spectrum’ with other conditions and with healthy experiences. They require the acceptance that patients do not present uniformly, and any attempts to shoehorn presentations into discrete entities are precarious.

An early protagonist of the dimensional view was Heinrich Neumann (1814–1884) who wrote that “*classification is only possible when there are genera, but these do not exist in the absence of’generation’* [aetiology]” [30]. Even Kraepelin’s enthusiasm to provide a neatly organised framework to Psychiatry was tempered after four decades of research. In 1922 he warned against the too rigid application of the system, acknowledging that there are numerous cases that share features of different categories [31]. Similarly, Eugen Bleuler in his seminal monograph *Dementia Praecox or the Group of Schizophrenias* ([16] p. 13) specified: “*it is extremely important to recognise that they [symptoms] exist in varying degrees and shadings in the entire scale from pathological to normal*”. Partly inspired by Bleuler’s ideas Sándor Radó suggested the existence of the latent variable of ‘schizotype’ [32] further elaborated into the concept of ‘schizotypy’ by Paul E. Meehl [33]. This was assumed to be reflective to a genetically acquired dysfunction of the brain, manifest along a quasi-linear spectrum as ‘cognitive slippage’. Interestingly, schizotypy made its way into the newest diagnostic systems as a separate diagnostic entity (i.e., schizotypal disorder), rather than an entity on a continuum with schizophrenia.

More recently dimensional models have been developed using factor analysis, identifying symptom clusters such as positive, negative and disorganisation symptom groups. The number of clusters varies between models, ranging from three [34] to five [35], or even seven [36]. Other models conceptualise psychosis on a continuum with healthy experiences and with affective disorders, incorporating external factors (such as psychological trauma, socio-demographic influences and substance use) forming the basis of bio-psycho-social formulations [37, 38]. These approaches have been boosted by a recent surge of studies demonstrating association between childhood psychological trauma and schizophrenia [39–41]. The impact of psychological trauma in psychosis has led to important theoretical models [42, 43] and even to the proposal of Traumatic Psychosis as a separate diagnostic entity [44].

Dimensional models of psychosis differ from each other in terms of what is it they envisage as having ‘dimensional’ character. Some of these models seek to maintain existing categories of our classification systems, while acknowledging the overlap between diagnoses (DSM-5 and ICD-11). In this sense they maintain a medical essentialist view, adopting a ‘soft-realist position’ regarding the ontological nature of diagnostic entities (as suggested by Kenneth Kendler [45]). Other approaches completely break away from existing diagnostic classifications, introducing ‘observables’ based either on a phenomenological or biological basis [46]. The former identifies phenomenological observables based on constitutive features of human experience and behaviour; an example of this would be the EASE model developed by Parnas et al., talking about cognition, stream of consciousness, self-awareness, bodily experiences, demarcation, existential orientation. [47]. The latter proposes the existence of ‘domains’ of human functioning as understood from the perspective of neuroscience, neurophysiology, genetics and experimental psychology, as in the six-domain model of the RDoc initiative [48].

Regarding dimensional approaches, it is often difficult to see how dysfunctions in different dimensions, as measured separately, end up occurring together to constitute disorders so often recognisable in clinical practice. Recent theoretical models try to address this dilemma by suggesting ways in which the whole can be more than the summation of its parts. An example of this is the ‘extended phenotype’ model of psychosis introduced by Jim van Os and Uli Reininghaus in 2016, recognising a general, trans-diagnostic psychosis factor and specific illness dimensions [49]. This model can also account for sub-threshold psychotic experiences that could be helpful to identify individuals with ‘at risk mental states’ for psychosis [50, 51]. A phenomenologist answer to the problem of dimensional atomisation has been proposed by Henriksen and Parnas suggesting that schizophrenia constitutes a certain psychopathological Gestalt of disturbed self-awareness, manifest in multiple experiential domains such as sense of identity, self-demarcation, self-organisation and belonging to the world [52].

### Network approaches

The most radical challenge of categorical and dimensional essentialist views has been brought about by the theory of complex systems, identifying mental disorder in ‘patterns of interactions’. This approach strongly embraces the idea of the ‘whole more than the sum of its parts’ and implies that mental disorders emerge dynamically as networks of complex systems interact with each other at different levels of organisation (biological, psychological, social). Although the elements of complex system theory can be found in numerous stages of philosophical thinking (Aristotle, Immanuel Kant, John Stuart Mill), unified models started to take ground in the 1970s with the work of Gregory Bateson, Albert-László Barabási, Murray Gell-Mann, Stuart Kaufman, and in psychopathology mainly by Denni Borsboom and his colleagues. The network approach can be used within the confines of one particular field (e.g., protein, neuron or symptom networks) or can be extended to include multiple systems (such as the six domains identified in the RDoc approach) and interactions with environmental variables (trauma, substance use, family interactions).

The network approach has been applied to psychosis in a relatively small number of studies [53]. Some of these papers explored interconnectedness between symptoms of psychosis [54, 55], between psychosis and other pathological experiences (such as depression, anxiety and distress) [56] and between psychotic symptoms and a history of trauma [57] and substance misuse [58].

Borsboom and colleagues propose a dynamic framework of mental disorders [59] in which the system moves from a ‘dispositional’, stable state into a ‘disturbed’ state (i.e., illness episode) precipitated by situational factors. The constitutive elements of the system interact with each other in reciprocal ways (feedback loops) forming self-sustaining patterns of interactions. In this model mental disorders are viewed in a soft-realist way, similarly to the way the reality of patterns is treated by Daniel C. Dennett, who states that “*A pattern exists in some data—is real—if there is a description of the data that is more efficient than the bit map* [verbatim description]” [60]. Without completely excluding the role of biological contributing factors Borsboom and colleagues reject the idea of biological explanatory reductionism, regarding mental disorders as intrinsically complex, born out of the very *process* of interactions [61]. In response to this statement Bringman and Eronen [62] argues that the network approach does not inherently contradict the biologically or psychologically oriented latent variable models. They suggest that strengths of the network models rely on providing a dynamic approach to psychopathology, offering insightful ways to visualise relationships and even to build mathematical models of them, but they do not change our knowledge of whether some factors could or could not be considered root causes of mental disorders. Indeed, the observation of superficial interactions between experiences (e.g., lack of sleep – altered reality perception – delusional ideation) does not rule out in principle the possibility of shared background causative factors.

Also, while the network approach can provide relatively plausible explanations for episodic disorders, it struggles to explain long-lasting phenomena such as developmental traits or longstanding negative and cognitive symptoms [59]. Another fundamental challenge of the network approach (similarly to factor analysis models) is the need to decide how broad should be the range of phenomena included in the network. Studies using the network approach need to have a broad and open-minded perspective, while being aware of the risk of making the analysis untenable by exponentially increasing complexity with increasing number of components.

### Learning from Cognitive Psychology and Linguistics

As we have seen above, regardless of our position about the ontological status of mental disorders, we create highly simplified mental representations of them based on our fundamental assumptions of their nature. This is where researchers’ and clinicians’ own psychological makeup comes into play, making their judgment vulnerable to Bacon’s idols [2].

The first of these, i.e. the ‘idol of the tribe’ is recognizable in our shared tendency to be drawn to the familiar, identify patterns and regard them with a greater or lesser degree of subjectivity, even emotional attachment. Also, the answers we get are always a function of the kind of questions we ask and the methods we use to investigate them (e.g., quantitative, qualitative or mixed design).

The ‘idols of the den’ in the clinical context might refer to the different priorities, predispositions and assumptions inherent to our professions of choice (e.g. Nurse, Psychiatrist, Psychologist, Social Worker etc.). This might influence for example, whether we are predominantly drawn to more biological, psychological or socially orientated theories of psychosis and opt for corresponding assessment and therapeutic approaches.

Perhaps the most subtle and problematic among the idols are the ‘idols of the market’ [2] which refer to the words we are using and the concepts they signify. Lexicalization (attaching labels to things) appears early in life by spontaneous exploration of the world [9] but also powerfully reinforced by parents or guardians [10]. Medin and Ortony [63] argue that psychological essentialism is a fundamental mechanism of representing objects of thought, and there is abundant evidence regarding the importance of object representation and constancy as steps of human cognitive development [10, 64, 65]. This process continues throughout our professional education, playing into the illusion that the concepts we internalise (e.g., diagnostic labels) possess some form of timeless, unchangeable ‘essential reality’, similar to Plato’s Forms or to mathematical axioms. However, as we have seen, the ontological status of mental disorders is not so straightforward.

Taking it one step further, thinking about how our concepts integrate in theoretical systems we can draw some important insights from the theories of schemas. The concept of ‘schema’ was first introduced by Immanuel Kant in 1971 in *Critique of Pure Reason* to describe the mediating procedural mental operations, or ‘rules’, connecting ‘pure concepts of understanding’ with the corresponding (manifold) concrete ‘objects of experience’. ([66], pp. 271–277). Much later ‘schema’ has become a fundamental concept in Cognitive Psychology through the work of Frederic Bartlett [67] (and many others later, including Piaget, Rumelhart, Minsky, Vygotsky) signifying patterns of declarative and procedural knowledge that are activated simultaneously, representing certain aspects of the world. These schemas aid recognition and facilitate mental economy, but also have an organising influence that can lead to selective attention, omission, and ‘fitting’ perception into pre-existing assumptions. Bartlett suggested that cognitive schemas are (primarily, but not exclusively) represented in the form of visual mental images as well as language. He also suggested that a schema can be generalised and transposed from one field to another, enabling the constructive imagination and thinking characteristic to human intellect. The concept of *schema* has been taken forward in Cognitive Linguistics by George Lakoff and Mark Johnson [68] in the form of the Conceptual Metaphor theory, describing how schemas (or, in their usage, image-schemas) become consolidated in linguistic expressions – conventional metaphoric expressions in everyday language, but also formalised expressions of philosophical and scientific constructs.

In the light of the above considerations, we would suggest that ‘clinical schemata’ are therefore linked to an internal schematic visual imagery as well as a set of collectively developed language, with its characteristic metaphoric mappings. In this sense the categorial approach seems to equate mental disorders with so many ‘bounded regions’, each ‘containing’ a ‘cluster’ of symptoms (individual ‘objects’). If we have sufficient ‘objects’ (even if these are different from case to case) then we assume that a certain diagnostic entity is present. This approach also invokes the metaphor of a physical building with a certain ‘architecture’, ‘building blocks’ and ‘thresholds’ in a complex hierarchical diagnostic system with mutually ‘exclusive’ categories (ICD-11, [1]). Using the dimensional approach, we might tend to employ the visual imagery of straight continuous lines, like a coordinate system, representing disorders on a ‘continuity’ with usual experience and symptoms as ‘dimensions’ of a disorder (ICD-11, [1]). It is also interesting how the most recent diagnostic systems are trying to reconcile the difference between these two views by talking about *“fluid boundaries between categories”* with *“[symptom] dimensions that cut across current [diagnostic] boundaries”* (DSM-V, Introduction [26]). Similarly, the network approach can lead us to envisage disturbances as ‘nodes’ of a ‘network’ and co-occurrences of symptoms as ‘edges connecting nodes of a network’, representing putative causal interactions.

We recognise that there are limitations to applying the Conceptual Metaphor theory to clinical thinking, as it is not always easy to find identifiable image schemas to clinical concepts. Also, by the above analysis we do not aim to dismiss the validity of the clinical frameworks discussed, as we recognise metaphoric representation as a “*basic functional aspect of the symbolisation process*” [47]. The reason why we are sharing these observations is that if we want to avoid falling victim to the ‘idols of the theatre’ [2], we need to bear in mind that these modes of thinking are all approximations, each with their characteristic biases. This awareness will enable us to avoid thinking of diagnostic categories a rigid ‘boxes’, of latent variables as ‘smooth uninterrupted lines’, and of ‘edges between nodes’ as simple, necessary and direct causal relationships.

## Conclusion

While unable to bring a definitive answer to the question of what exactly psychosis is, in this paper we attempted to trace back the development of the concept and look at three different ways it is formulated. While the three approaches discussed in this paper are relatively separated within research settings, they are inseparably intertwined in the clinicians’ mind during the process of taking a history, proposing diagnoses, and creating formulations. It seems that our visual imaginative faculty plays an important part in this process, as we tend to envisage disorders as clusters, lines or webs and subsequently use a metaphoric symbolisation process to describe them. We linked our discussion to Francis Bacon’s epistemological writings and to considerations regarding the ontological status of mental health diagnosis. We brought into discussion theories from Cognitive Psychology and Linguistics to emphasize the subjective nature of these frameworks and to highlight the importance of clinicians’ awareness of their strengths and limitations. In our view, this will help to avoid the dangers of dogmatic, unilateral thinking and will make clinicians less vulnerable to the ‘idols’ of their minds. The answer to our initial question about the nature of psychosis will then develop in the process of individual understanding of each person’s difficulties within a multilateral and collaborative process; although this will never work well with rigid care pathways and inflexible guidelines.

## Data Availability

Not applicable.
